# Research on the Method of Optimizing the Stress and Improving the Performance for MEMS Gyroscope Based on the Cantilever-Plate Structure

**DOI:** 10.3390/mi16040372

**Published:** 2025-03-25

**Authors:** Yunbin Kuang, Xiaoyan Huo, Weitao Guo, Xiaoxing Li, Jiangyan He, Qiong Mao, Xiaolin Ma, Jie Liu

**Affiliations:** 1 Shijiazhuang Campus, Army Engineering University of PLA, Shijiazhuang 050003, China; kuangyunbin16@nudt.edu.cn (Y.K.);; 2 31638 Units of PLA, Kunming 650034, China

**Keywords:** MEMS gyroscope, thermal stress, cantilever plate structure, bias hysteresis, frequency drift

## Abstract

Thermal stress is one of the most important factors damaging the temperature-dependent performance of MEMS gyroscopes. To reduce thermal stress and improve their performance, this paper deduced the production and effects of thermal stress on a high-precision MEMS butterfly gyroscope theoretically, which provided a basis for optimization and experiments. A novel cantilever plate structure was designed based on the working modes of the MEMS butterfly gyroscope and optimized based on our simulation to achieve stress isolation. The simulation results showed that after integrating the cantilever plate structure, the stress acting on the MEMS butterfly gyroscope was reduced by 346 times, while the average capacitance gap error was also reduced by 36 times within the same variable temperature range. In addition, the cantilever plate structure was fabricated and integrated with the MEMS butterfly gyroscope. Experiments were also conducted to demonstrate the effect of reducing the thermal stress, and the results showed that the frequency variation was reduced by 28.6% and the bias stability increased by about 2 times over the full temperature range after integrating the cantilever plate structure into the gyroscope. This demonstrated that the cantilever plate structure can effectively reduce thermal stress and improve the performance of the MEMS butterfly gyroscope.

## 1. Introduction

Micro-electromechanical system (MEMS) gyroscopes have received a lot of attention in inertial navigation systems due to their miniaturization, low cost, high reliability, batch fabrication, and IC (integrated circuit)-compatible integration [1,2,3,4]. With the optimization of this technology’s designed structures, signal detection, and fabrication, the constant-temperature performance of MEMS gyroscopes has greatly improved, gradually leading to navigation-grade performance [5,6,7,8,9].

However, most MEMS gyroscopes are applied in variable temperature environments. The thermal stress produced by temperature variations causes their working frequency, damping, and capacitance gap to change, which results in bias error. Moreover, the hysteresis of the strain produces different outputs from the heating and cooling processes, which means that the compensation algorithm cannot eliminate the error completely and the improvement in the temperature-dependent performance of the gyroscope is limited [10].

In terms of optimizing the stress on MEMS gyroscopes, many methods have been proposed in recent years. To sum these up, they can be mainly divided into three categories: process optimization, structure optimization, and stress isolation. The University of Chinese Academy of Sciences studied residual internal stress and packaging stress in gyroscopes and reduced both by optimizing the annealing process before and after the gyroscope was packaged.After process optimization, the drift time and amount of bias in its normal-temperature starting stage are reduced by 1.7 and 2.2 times, respectively [11]. The Hitachi Corporation of Japan designed a single-sided open driving structure for a micro-tuning fork gyroscope to suppress the influence of thermal stress [12]. After simulating and analyzing the deformation of four-mass micro-gyroscope caused by thermal stress, Tsinghua University designed a thermal deformation suppression chip to reduce the structural deformation caused by thermal stress. After stress isolation, the deformation of the MEMS gyroscopes’ structure is reduced by about 10 times compared to the original deformation [13].

Compared with the methods that optimize the process and optimize the structure, stress isolation is more convenient and reduces the effect of thermal stress on MEMS gyroscopes more easily. Therefore, in this paper, we focus on a kind of high-precision x/y-axis MEMS gyroscope called a butterfly gyroscope and aim to reduce its thermal stress by performing a production analysis of that stress, designing and optimizing a cantilever plate structure, and fabricating and integrating the cantilever plate structure into the gyroscope. Firstly, the production and effect of thermal stress are analyzed theoretically, which provides us with a basis for optimization and performing experiments. Then, a cantilever plate structure is proposed and designed to isolate thermal stress according to the working principle and modes of the MEMS butterfly gyroscope. The critical sizes of the cantilever plate structure are also optimized by simulation. After fabrication and integration, experiments are conducted and the results show that the frequency drift is reduced by 28.6%, the bias hysteresis is decreased by 2 times, and the temperature-dependent bias stability is also improved from 26.27°/h to 9.7°/h. The experimental results demonstrate the effect of stress isolation and this paper also provides an effective method for greatly improving the temperature-dependent performance of the MEMS butterfly gyroscope.

## 2. Theoretical Effect of Thermal Stress in the MEMS Butterfly Gyroscope

### 2.1. Structure and Working Principle of the MEMS Butterfly Gyroscope

**Figure 1:**
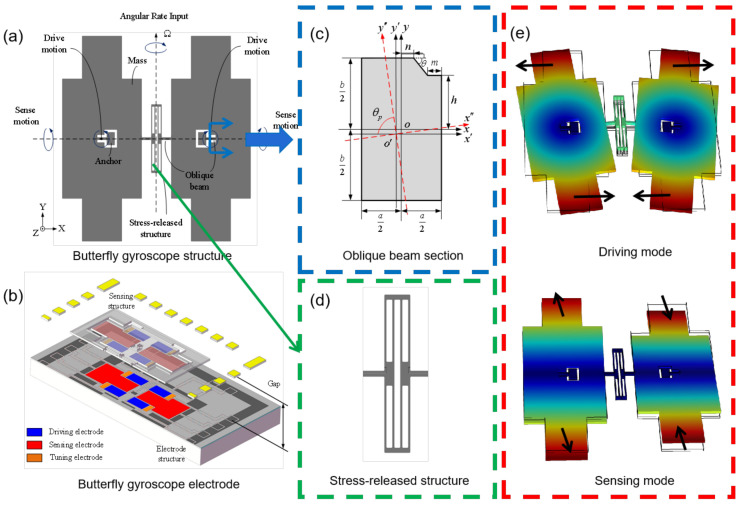
The structure of the MEMS butterfly gyroscope: (**a**) the sensing structure; (**b**) the section view of the three-dimensional structure; (**c**) the cross-section of the novel oblique vibration beam; (**d**) the stress relief structure; (**e**) the working modes including driving mode and sensing mode.

The mechanical structure of the MEMS butterfly gyroscope is shown in Figure 1a. It contains two masses connected by a vibration beam. Two anchors inside the masses are bonded to the substrate with electrodes [6,14,15,16]. The cross-section view of the three-dimensional structure of the butterfly gyroscope is shown in Figure 1b. The masses and their corresponding electrodes form a couple of capacitances that drive the mechanical structure and detect structure variations. The capacitance gap is 2 μm. As shown in Figure 1b, three types of electrodes are designed: a driving electrode, sensing electrode, and tuning electrode.

The vibration beams are the most critical part in the butterfly gyroscope, and they are designed to be an oblique beam, as shown in Figure 1c. As the spindle azimuth is not 90°, the normal force from the driving electrode will produce a tangential component to drive the mass to vibrate in the plane, like Figure 1e demonstrates. The drive mode usually operates using self-resonance to minimize the excitation voltages and achieves a stable amplitude and phase. With an angular rate input, there will be a sinusoidal oscillation induced by the Coriolis force in the sensing direction, as is shown in Figure 1e. Therefore, the angular rate input can be measured by detecting the capacitance variation in the sensing direction through the sensing electrode.

Compared to the original butterfly gyroscope structure designed by Sensonor [17], the proposed structures are the oblique beam and the stress relief structure shown in Figure 1c,d. Due to the normal-direction displacement of the driving mode, the gap will limit the amplitude in the tangential direction, which restricts the improvement of the butterfly gyroscope’s sensitivity. It is proposed to fabricate a hexagon cross-section beam by wet etching and DRIE process to improve the spindle azimuth so as to improve the driving amplitude. In addition, the stress relief structure was designed in the middle vibration beam. It can reduce the influence of thermal stress on the vibration beam and keep the driving motion symmetrical.

### 2.2. Theoretical Effect of Thermal Stress on the MEMS Butterfly Gyroscope

Thermal stress varies with temperature, so the inherent characteristics of the MEMS butterfly gyroscope also change with temperature under the influence of thermal stress. The impact is mainly manifested in two aspects. On the one hand, the axial stress it generates acts on the vibrating cantilever of the MEMS butterfly gyroscope, leading to a temperature-dependent drift in the gyroscope’s modal frequency. On the other hand, it causes deformation of the electrode layer and the sensitive structural layer of the MEMS butterfly gyroscope, resulting in temperature-dependent changes in capacitance gap.

**Figure 2:**
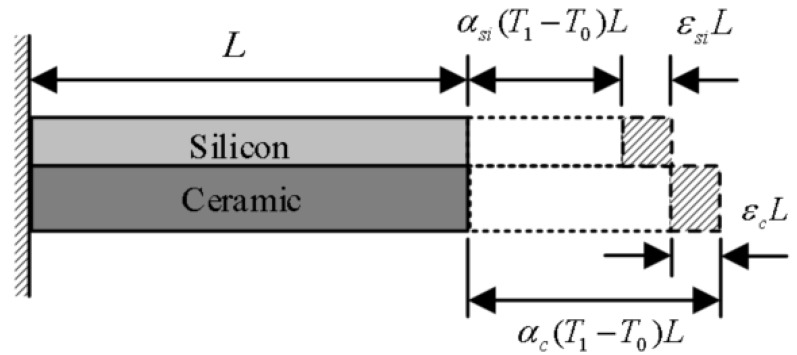
The simplified model for the analysis of the thermal stress.

The vibratory beam of the MEMS butterfly gyroscope is directly connected to the electrode layer by the anchor. And compared with the electrode layer, the width of the beam is much smaller, so it can be considered that the stress of the beam is the same as that of the electrode layer. The stress of the electrode layer is mainly due to the mismatch of thermal expansion between silicon and the ceramic envelope. Therefore, the model of thermal stress for the MEMS butterfly gyroscope can be simplified as shown in Figure 2.

Assume that there is no constraint between silicon and the ceramic envelope, and the temperature is stable. The expansion displacement of the silicon vibration beam and the ceramic envelope can be deduced as follows.

(1) $$\Delta L_{si}=\alpha_{si}\left(T_{1}-T_{0}\right)L$$

(2) $$\Delta L_{c}=\alpha_{c}\left(T_{1}-T_{0}\right)L$$

$\Delta L_{si}$ and $\Delta L_{c}$ are the thermal expansion displacement of the silicon vibration beam and the ceramic envelope, respectively. $\alpha_{si}$ and $\alpha_{c}$ are their coefficients of thermal expansion. $T_{0}$ represents the initial temperature, and *L* represents the initial length. The lengths $\epsilon_{c}L$ and $\epsilon_{s_{i}}L$ express the deviation of the thermal expansion displacement in rigidly bonded silicon and ceramic materials from their free thermal expansion states, resulting from a mismatch in the thermal expansion coefficients under thermal loading.

In fact, the vibration beam fixedly connects with the ceramic envelope. Due to the mismatch of thermal expansion, the stress is produced between them. Assuming that there is no warping between the silicon vibration beam and the ceramic envelope, and no external force is applied, the internal stress of them is equal. The stress of the vibration beam can be expressed as follows.

(3) $$\sigma_{si}=\frac{\alpha_{si}E_{si}\left(T_{1}\right)\left(T_{1}-T_{0}\right)\left(1-\frac{\alpha_{si}}{\alpha_{c}}\right)}{1+\frac{A_{si}E_{si}\left(T_{1}\right)}{A_{c}E_{c}\left(T_{1}\right)}}$$

$E_{si}\left(T_{1}\right)$ and $E_{c}\left(T_{1}\right)$ are the Young’s modulus of silicon and ceramic, respectively. $A_{si}$ and $A_{c}$ are the cross-sectional area of the silicon vibration beam and the ceramic envelope. Because $A_{si}<<A_{c}$, Equation (3) can be simplified as

(4) $$\sigma_{si}\approx \alpha_{si}E_{si}\left(T_{1}\right)\left(T_{1}-T_{0}\right)\left(1-\frac{\alpha_{si}}{\alpha_{c}}\right)$$

The axial stress of the silicon vibration beam can be expressed as

(5) $$N_{si}=\sigma_{si}A_{si}\approx A_{si}\alpha_{si}E_{si}\left(T_{1}\right)\left(T_{1}-T_{0}\right)\left(1-\frac{\alpha_{si}}{\alpha_{c}}\right)$$

Ceramic and silicon plates are firmly bonded together, and in fact, there is also thermal deformation between them. For the MEMS butterfly gyroscope, the driving frequency is predominantly governed by the axial stress of silicon vibrating beams. The thermal deformation between silicon and ceramic substrates exhibits negligible influence on the driving frequency, and is therefore not discussed in the theoretical analysis. It is deduced that the influence of axial stress on the driving frequency of the MEMS butterfly gyroscope can be expressed as

(6) $$f_{d}=f_{0}\sqrt{1+\frac{9N_{si}L^{2}}{\pi^{2}E_{si}\left(T_{1}\right)I_{d}}}$$

$f_{0}$ is the initial driving frequency, and $I_{d}$ is the moment of inertia of driving mode.

Since the thermal expansion coefficient of silicon is smaller than that of ceramic, according to Equations (5) and (6), the axial stress of the vibratory beam is tensile stress when the temperature increases, which means $N_{si}>0$, so that the driving mode frequency of the MEMS butterfly gyroscope will also increase. Similarly, the driving mode frequency will reduce when the temperature decreases. The gyroscope’s driving mode frequency will also drift with the temperature due to the change of Young’s modulus [18], so the effect of axial stress is reflected in amplifying the drift of the driving mode frequency in the same variable temperature range. This conclusion can be used to evaluate the stress of the MEMS butterfly gyroscope in the following experiments.

Thermal stress also causes the structural deformation of the MEMS butterfly gyroscope, which will result in the bias error. The MEMS butterfly gyroscope can be regarded as a second-order spring mass damping system. Fabrication errors are inevitable and induce the main three types of errors in dynamics: stiffness asymmetry, damping asymmetry and force asymmetry. And the closed-loop control of the sense mode is usually adopted to improve the stability of the high-precision MEMS gyroscope. Considering all the errors, the model of the butterfly gyroscope can be built. For the second-order dynamics system, the method of rotating-wave approximation can be applied. The bias output $Q_{Bias}$ and the scale factor SF of the butterfly gyroscope can be derived as follows. The detailed process of deduction can be referred to in [19].

(7) $$Q_{Bias}=\frac{K_{A}}{2\sin\theta_{p}}\left[\frac{2}{\tau }\frac{I_{x}}{I_{y}}\alpha_{1}-\Delta \left(\frac{1}{\tau }\right)\sin2\theta_{\tau }\right]*SF$$

(8) $$SF=2I_{x}x_{0}\omega_{d}$$

where

$$\begin{array}{cc} 2/\tau =1/\tau_{x}+1/\tau_{y} & \Delta 1/ \end{array}\tau =1/\tau_{x}-1/\tau_{y}$$

Among them, $K_{A}$ is the gain of the control system, $I_{x}$ and $I_{y}$ are the efficient mass, $\theta_{p}$ is the spindle azimuth, $x_{0}$ is the driving amplitude, $\alpha_{1}$ is the angle error of the driving force. They usually remain constant in the closed-loop control mode. $\omega_{d}$ represents the driving frequency. $\theta_{\tau }$ represents the angle error of the damping asymmetry. $\tau_{x}$ and $\tau_{y}$ are the decay time of the driving mode and the sensing mode, respectively, which are related to the damping.

For the MEMS butterfly gyroscope, the angle errors of the driving force are usually ignored and the decay time of the sensing mode is more than 100 times that of the driving mode. Therefore, the bias of the MEMS butterfly gyroscope $\Omega_{bias}$ can be simplified as follows.

(9) $$\Omega_{bias}=-\left(\frac{1}{\tau_{x}}-\frac{1}{\tau_{y}}\right)\sin2\theta_{\tau }\approx \frac{1}{\tau_{y}}\sin2\theta_{\tau }$$

According to Equation (9), the bias output of the MEMS butterfly gyroscope mainly depends on the damping of the sensing mode. When the squeeze-film damping of the sensing mode changes, the bias output of the gyroscope also changes accordingly. The coefficient of the squeeze-film damping is inversely proportional to the cube of the capacitance gap [20]. Therefore, the capacitance gap error caused by thermal stress in a variable temperature environment has a significant impact on the drift of the gyroscope’s bias output. Moreover, the strain caused by thermal stress in the gyroscope has hysteresis characteristics [21], which results in different capacitance gap errors at the same temperature point during the heating and cooling process. The bias drift curves are shown to be non-overlapping during the heating and cooling process, which is called bias hysteresis. This error cannot be eliminated by using the usual compensation algorithm. The complex compensation algorithm is required to reduce the error, which not only occupies more computational resources but also has extremely high requirements for the repeatability of the gyroscopes’ bias output [22]. The effect is also unsatisfactory after compensation. Therefore, reducing thermal stress is an effective way to suppress the bias hysteresis and enhance the performance of the MEMS butterfly gyroscope over the full temperature range.

## 3. Design and Optimization of the Cantilever Plate Structure

### 3.1. Design of the Cantilever Plate Structure

**Figure 3:**
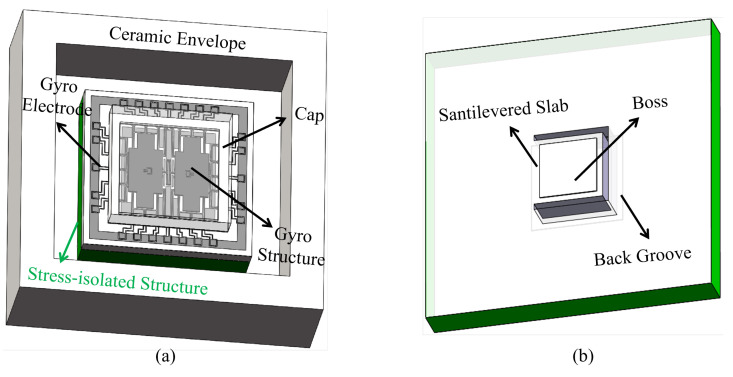
(**a**) shows all the components of the vacuum packaged butterfly gyroscope after integrating the cantilever plate structure. (**b**) shows the components of the cantilever plate structure.

Based on the vibratory shape of the MEMS butterfly gyroscope, the cantilever plate structure is proposed and designed to isolate stress, which is shown in Figure 3. In order to reduce the influence of the thermal stress, the structure is displaced between the ceramic envelope and the gyroscope. The bottom of the structure is attached to the substrate of the ceramic envelope through conductive adhesive, and the top is bonded with the gyroscope through the boss of the cantilever plate, as shown in Figure 3a. The main body of the cantilever plate structure is a cantilever plate fabricated by DRIE (Deep Reaction Iron Etching) on the silicon substrate. In order to make the gyroscope only contact the cantilever plate structure, a boss is designed on the cantilever plate. And a groove is designed on the back of the cantilever plate structure to provide the deformation space for the cantilever plate. The detailed cantilever plate structure is shown in Figure 3b.

Without the cantilever plate structure, the thermal stress is directly transferred from the ceramic envelope to the gyroscope, which will cause deformation of the sensitive structure. The principle of the cantilever plate structure is to transfer the stress to the cantilever plate. Due to the low normal stiffness, the cantilever plate will be bent under the action of thermal stress, so as to achieve stress relief. At this time, the gyroscope chip will be moved as a whole with the deformation of the cantilever plate, but the deformation of its internal structure, especially the capacitance gap, will be greatly reduced.

### 3.2. Optimization of the Critical Sizes

In order to obtain a better effect of stress isolation, this paper optimizes the critical sizes of the cantilever plate structure by simulation. The key sizes of the cantilever plate structure include the length of the cantilever plate structure, the thickness of the cantilever plate structure, the length of the cantilever plate, the thickness of the cantilever plate, the length of the boss and the height of the boss.

In order not to increase the area of the gyroscope, the length of the cantilever plate structure is designed to be consistent with that of the gyroscope chip (5 mm). In addition, when the cantilever plate structure is thicker, it is obvious that the thermal stress transferred to the gyroscope becomes less. But due to the limit of the ceramic envelope’s height, the thickness of the cantilever plate structure can only be designed for a maximum of 800 µm. In addition, the function of the boss is to make the gyroscope contact with the cantilever plate. In order to reduce its effect on the modal frequency and simplify the simulation of the design, the height of the boss is designed to be as small as 10 µm at the processing capacity. According to the symmetrical structural features of the MEMS butterfly gyroscope, the shape of the boss is designed to be a square. The length of the boss is designed to be 100 µm smaller than that of the cantilever plate, which not only plays the role of the boss, but also makes it so that the influence of the boss on the stress and the modal frequency can be ignored, which is convenient for the analysis and design of other critical sizes. Therefore, this paper focuses on the length and thickness of the cantilever plate, and achieves their size optimization by simulation.

**Figure 4:**
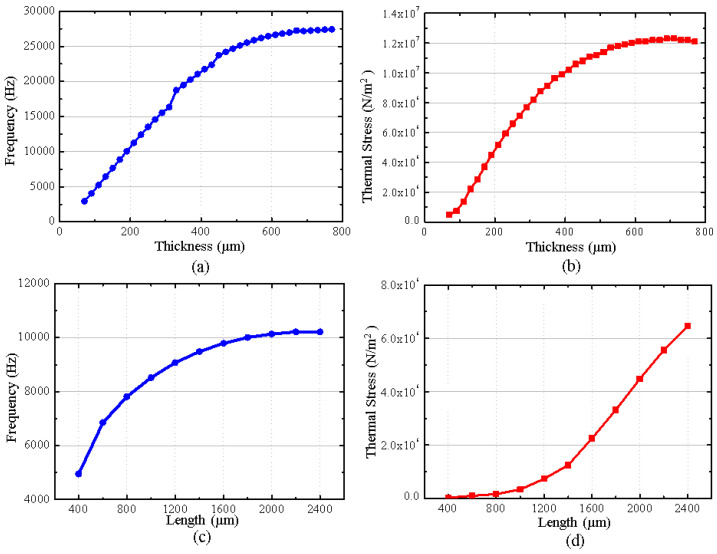
(**a**) shows the influence regularity of the cantilever plate’s thickness on the resonant frequency. (**b**) shows the influence regularity of the cantilever plate’s thickness on the thermal stress. (**c**) shows the influence regularity of the cantilever plate’s length on the resonant frequency. (**d**) shows the influence regularity of the cantilever plate’s length on the thermal stress.

In this simulation, the thermal stress and resonant frequency are the key. The effects of the cantilever plate’s thickness and length on the resonance frequency and thermal stress are simulated as shown in Figure 4.

As shown in Figure 4a,b, when the length of the cantilever plate remains unchanged and the thickness increases, the resonant frequency and the thermal stress increase. And their influence regularity is almost the same, which shows that as the thickness increases, the growth rate of the resonant frequency and thermal stress will slow. It demonstrates that the influence of the cantilever plate’s thickness on thermal stress completely depends on the resonance frequency. The lower the resonance frequency, the better the effect of the thermal stress releases.

As shown in Figure 4c, when the thickness of the cantilever plate remains unchanged and the length increases, the resonant frequency increases and its growth rate shows a gradual decrease. However, the regularity of the thermal stress affected by the length of the cantilever plate is different. As shown in Figure 4d, although the thermal stress also increases with the increase in the cantilever plate’s length, its growth rate shows a gradual increasing trend. It demonstrates that the thermal stress of the MEMS butterfly gyroscope is affected not only by the resonant frequency, but also by the length of the cantilever plate due to the shape and size of the gyroscope. According to the simulation results, it can be concluded that for the structure of the MEMS butterfly gyroscope, the smaller length of the cantilever plate can bring about a better effect of releasing thermal stress under the condition of the same resonant frequency.

The resonant frequency of the MEMS butterfly gyroscope is about 6 kHz. In order to prevent the vibratory impact of the cantilever plate on the gyroscope, its resonant frequency should be kept away from the resonant frequency of the gyroscope. Based on the influence regularity of the simulation, the lowest vibratory mode frequency of the cantilever plate structure is designed to be about 10 kHz, which can not only reduce the influence on the operating mode of the gyroscope, but also provide a better effect of the thermal stress release.

**Figure 5:**
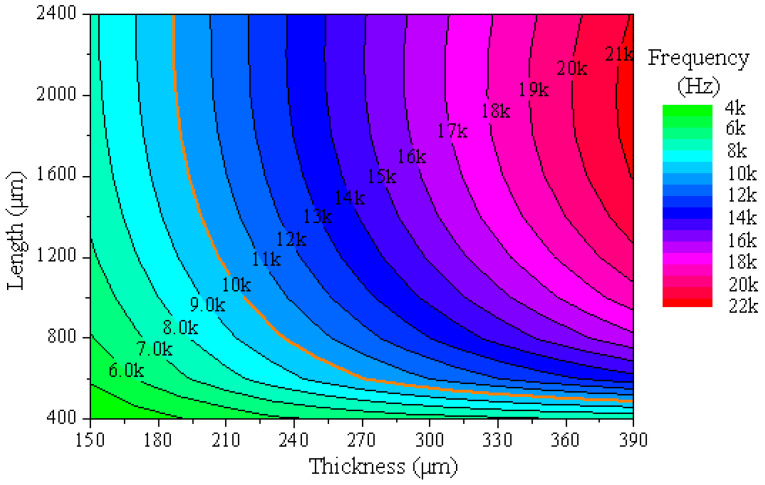
The simulation results of the resonant frequency under the condition of different length and thickness of the cantilever plate.

The vibratory frequency of the cantilever plate structure under the condition of different length and thickness is simulated, as shown in Figure 5.

It can be seen that when the thickness of the cantilever plate is less than 190 µm, the resonant frequency is lower than 10 kHz. When the length of the cantilever plate is less than 500 µm, the resonant frequency is also less than 10 kH. In addition, according to the analysis of the simulation result, the thermal stress on the MEMS butterfly gyroscope is less when the length of the cantilever plate is smaller at the same resonant frequency. Combined with the ability of fabrication, the cantilever plate’s thickness and length are designed to be 270 µm and 600 µm, respectively, and the resonant frequency is 9989 Hz.

### 3.3. Simulation of the Stress Isolated Effect

**Figure 6:**
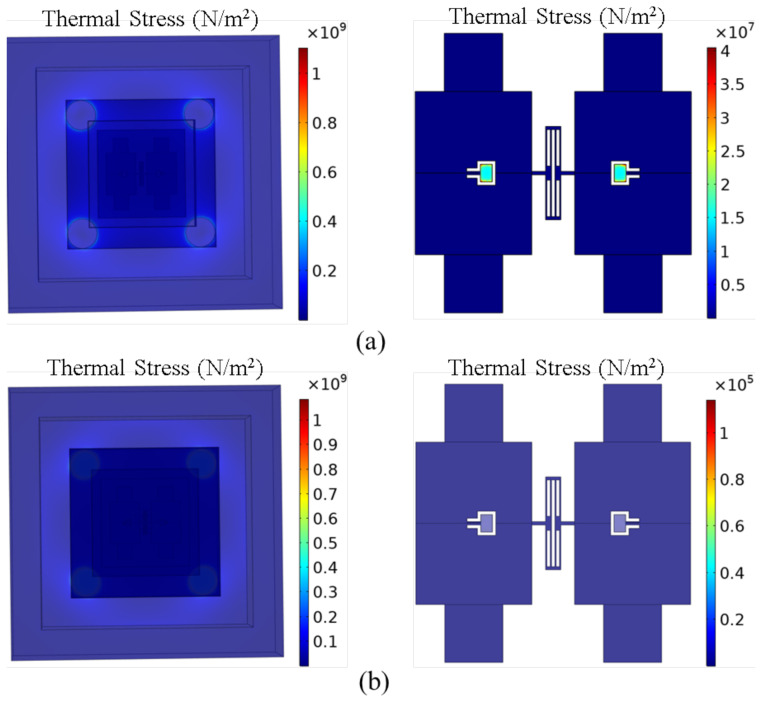
(**a**) The simulation result of the thermal stress in the MEMS butterfly gyroscope before integrating the cantilever plate structure. (**b**) The simulation result of the thermal stress in the MEMS butterfly gyroscope after integrating the cantilever plate structure.

In order to verify the suppression effect of the cantilever plate structure on the thermal stress, the thermal stress of the MEMS butterfly gyroscope is simulated before and after integrating the cantilever plate structure. The simulation results are shown in Figure 6.

It can be seen that under the same condition of temperature variations, the total thermal stress is the same before and after integrating the cantilever plate structure, both being $1.1\times 10^{9}\;{N/m}^{2}$. However, the maximum stress on the gyroscope is quite different. Without the cantilever plate structure, the maximum stress on the gyroscope is about $4.4\times 10^{7}\;{N/m}^{2}$, while the maximum stress on the gyroscope is only about $1.3\times 10^{5}\;{N/m}^{2}$ after integrating the cantilever plate structure, which is 346 times less than before.

**Figure 7:**
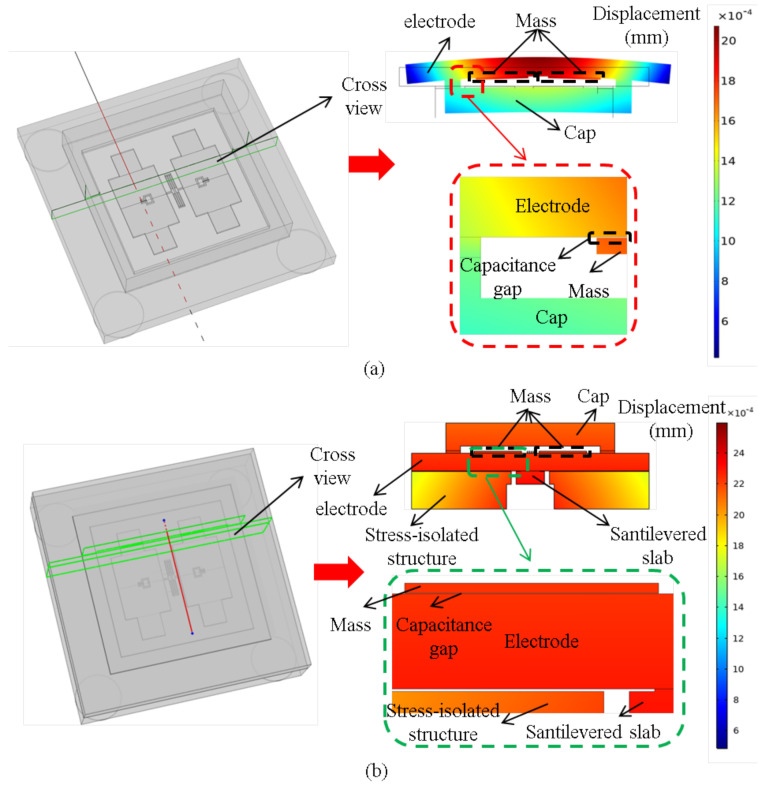
(**a**) The simulation result of the capacitance gap error in the MEMS butterfly gyroscope before integrating the cantilever plate structure. (**b**) The simulation result of the capacitance gap error in the MEMS butterfly gyroscope after integrating the cantilever plate structure.

Further simulations were conducted on the capacitance gap error of the MEMS butterfly gyroscope before and after integrating the cantilever plate structure over the full temperature range. The normal cross-sectional view and the simulation results of normal displacement are shown in Figure 7. By comparing the simulation results of the capacitance gap error, it can be observed that although the normal displacement over the full temperature range increases due to the deformation of the cantilever plate after integrating the cantilever plate structure, the capacitance gap error is significantly reduced. The simulation results indicate that the average capacitance gap error over the full temperature range is only 0.0029 µm, which is reduced by 36 times compared to the original value. In summary, the optimized cantilever plate structure can effectively reduce the impact of thermal stress on the MEMS butterfly gyroscope.

## 4. Fabrication and Performance Tests of the Cantilever Plate Structure

### 4.1. Fabrication and Integration

**Figure 8:**
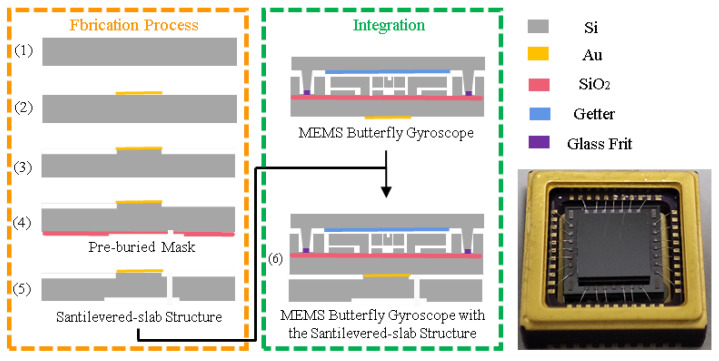
The fabrication and integration process of the cantilever plate structure.

Figure 8 shows the schematic views of the fabrication and integration of the cantilever plate structure. The detailed process flow is introduced as follows.

(1) A silicon wafer is prepared for fabricating the cantilever plate structure.

(2) Sputter a gold layer on the front side of the silicon wafer and pattern it at the corresponding positions of the small bosses for bonding with the gyroscope chip.

(3) DRIE (Deep Reaction Iron Etching) is adopted to fabricate the small boss.

(4) Adopt the pre-buried mask process on the back side of the silicon wafer, which involves using photoresist coating and multiple photolithography patterning processes to form two layers of etching masks with different thicknesses on the back side of the silicon wafer.

(5) Based on the pre-buried masks, perform the DRIE to simultaneously achieve two different etching depths, obtaining the back recess structure while releasing the cantilever plate structure.

(6) Bond and integrate the cantilever plate structure with the MEMS butterfly gyroscope.

### 4.2. Performance Tests

**Figure 9:**
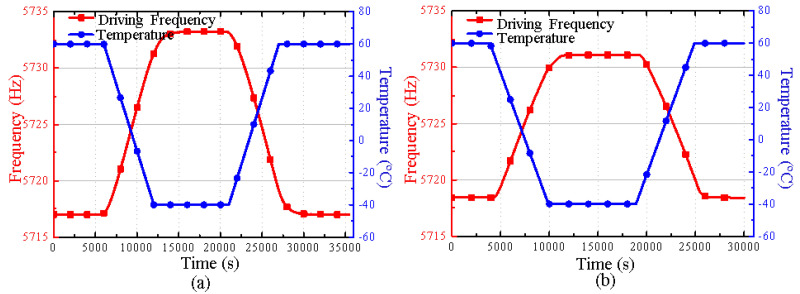
(**a**) The experimental result of the driving mode frequency drift before integrating the cantilever plate structure. (**b**) The experimental result of the driving mode frequency drift after integrating the cantilever plate structure.

According to the theoretical analysis, the influence degree of the thermal stress on the gyroscope can be reflected by testing the drift of the driving frequency at variable temperatures. The experiment is conducted, where the temperature range is set from −40 °C to 60 °C, and the change rate is set at 1 °C/min. The experimental results are shown in Figure 9.

It can be seen that the driving frequency decreases as temperature increases. Figure 9a shows that without integrating the cantilever plate structure, the driving frequency changes from 5733.3 Hz to 5717 Hz in the temperature range from −40 °C to 60 °C, and the driving frequency drifts by 16.3 Hz. After integrating the cantilever plate structure, the driving frequency changes from 5731.1 Hz to 5718.4 Hz in the same temperature range, and the driving frequency drifts by 12.7 Hz, which is shown in Figure 9b. The frequency variation is reduced by 28.6%.

**Figure 10:**
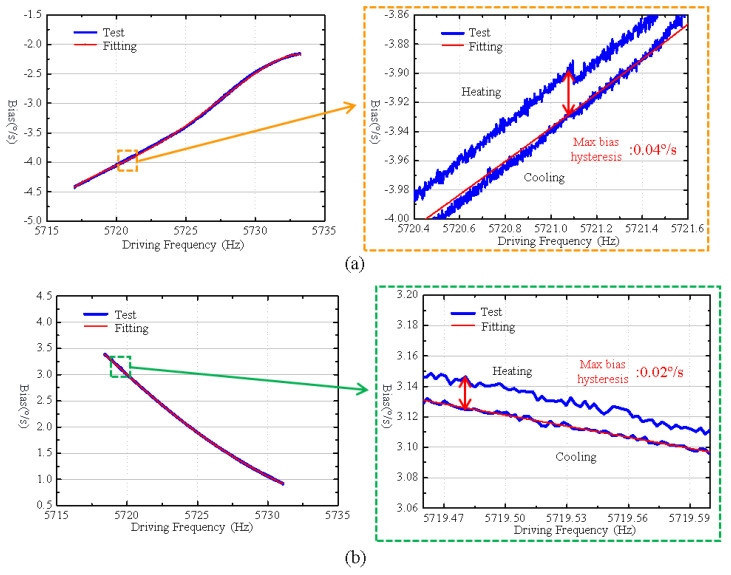
(**a**) The experimental result of the bias output before integrating the cantilever plate structure. (**b**) The experimental result of the bias output after integrating the cantilever plate structure.

The performance tests of gyroscopes are conducted in the temperature range from −40 °C to 60 °C; the measured driving frequencies, bias outputs, and their polynomial fitting curves are shown in Figure 10. It reveals that after integrating the cantilever plate structure, the maximum bias hysteresis error during the heating and cooling process is 0.02°/s, which is halved compared to the same gyroscope without the cantilever plate structure.

**Figure 11:**
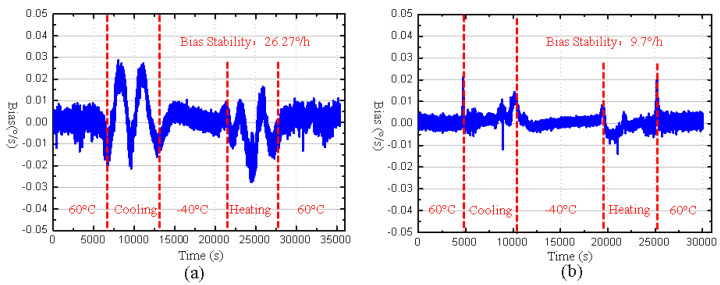
(**a**) The bias output of the MEMS butterfly gyroscope without the cantilever plate structure after compensation. (**b**) The bias output of the MEMS butterfly gyroscope with the cantilever plate structure after compensation.

The experimental bias outputs of two gyroscopes in the full temperature range shown in Figure 10 are compensated by using a fourth-order polynomial fitting, and the compensated results are shown in Figure 11. It can be observed that the maximum bias drift of the MEMS butterfly gyroscope without the cantilever plate structure reached 0.054°/s, while the maximum bias drift of the gyroscope with the cantilever plate structure is only 0.022°/s, which is halved compared to the former. Meanwhile, the bias stability of the MEMS butterfly gyroscope without the cantilever plate structure in the full temperature range is 26.27°/h, while that of the MEMS butterfly gyroscope with the cantilever plate structure is 9.7°/h, achieving a performance improvement of approximately 2 times.

For verifying the effect of the cantilever plate structure, 10 specimens are fabricated and tested. Experimental results including driving frequency, bias hysteresis error, and bias stability are shown in Table 1. All the experimental results show a similar conclusion that the driving frequency drifts and the bias hysteresis error are smaller, and the temperature-dependent bias stability has been improved after the cantilever plate structure has been integrated. It verified the robustness of the approach and the effect of reducing thermal stress and improving the performance of the MEMS gyroscope.

### 4.3. Analysis of Experimental Results

According to the theoretical analysis, thermal stress will amplify the drift of the driving mode frequency in the same variable temperature range. And the experimental results show that the variation of the driving frequency is reduced, which means that the cantilever plate structure can effectively reduce the thermal stress on the MEMS butterfly gyroscope.

Furthermore, thermal stress not only leads to the drift of the driving frequency, but also, due to the stress hysteresis, it causes bias hysteresis of the MEMS butterfly gyroscope over the full temperature range. It damages the bias stability of the MEMS gyroscope over the full temperature range. The experimental results show that the maximum bias hysteresis error of the butterfly gyroscope decreases and the bias stability improves after the cantilever plate structure is integrated. It also demonstrates the effect of the cantilever plate structure of reducing thermal stress and improving performance for the gyroscope.

## 5. Conclusions

This paper focuses on thermal stress, which is an important factor damaging the temperature-dependent performance of the MEMS butterfly gyroscope. The theoretical effect of the thermal stress is deduced. It reveals that thermal stress will amplify the frequency drift and the bias hysteresis in the same variable temperature range, which can be used to evaluate the stress of the MEMS butterfly gyroscope in the following experiments. To reduce the thermal stress, a novel cantilevered plate structure is designed according to the working modes of the MEMS butterfly gyroscope. And the critical sizes are optimized based on simulation results and the actual fabrication. The simulation results show that stress and its induced capacitance gap error of the gyroscope are greatly reduced in the same variable temperature range after integrating the optimized cantilever plate structure. Performance tests of the gyroscope are also conducted after fabricating and integrating the cantilever plate structure, which shows that thermal stress decreases and the bias stability increases by about 2 times over the full temperature range. It demonstrates that the cantilever plate structure can effectively reduce thermal stress and improve the performance for the MEMS butterfly gyroscope.

## Figures and Tables

**Table 1:** The experimental results of the MEMS butterfly gyroscopes with and without the cantilever plate structure.

Samples	Driving Frequency (Hz)	Frequency Drift (Hz)	Maximum Bias Hysteresis Error (°/s)	Bias Stability (°/h)
No. 1 (original)	5733.3–5717	16.3	0.054	26.27
No. 1 (improved)	5731.1–5718.4	12.7	0.022	9.7
No. 2 (original)	5725.5–5710	15.5	0.097	42.13
No. 2 (improved)	5723.1–5711.6	12.5	0.052	24.21
No. 3 (original)	5711.3–5692.5	18.8	0.11	45.32
No. 3 (improved)	5709.1–5695.9	13.2	0.08	33.56
No. 4 (original)	5730.6–5713.1	17.5	0.068	29.03
No. 4 (improved)	5728.2–5714.4	13.8	0.035	15.3
No. 5 (original)	5729.2–5713.4	15.8	0.074	31.25
No. 5 (improved)	5727.1–5714.7	12.4	0.04	16.17
No. 6 (original)	5728.8–5712	16.8	0.065	28.72
No. 6 (improved)	5726.1–5713	13.1	0.037	14.7
No. 7 (original)	5726.3–5710.5	15.8	0.09	35.24
No. 7 (improved)	5724.7–5711.1	13.6	0.055	27.63
No. 8 (original)	5735.2–5718	17.2	0.058	28.56
No. 8 (improved)	5733.6–5720.2	13.4	0.03	12.62
No. 9 (original)	5733.7–5718	15.7	0.075	31.68
No. 9 (improved)	5731.9–5719.4	12.5	0.039	14.83
No. 10 (original)	5736.9–5719.7	17.2	0.085	32.56
No. 10 (improved)	5734.5–5721.1	13.4	0.041	16.79

## Data Availability

The original contributions presented in this study are included in the article. Further inquiries can be directed to the corresponding author.
